# Artificial Intelligence Powered Automated and Early Diagnosis of Acute Lymphoblastic Leukemia Cancer in Histopathological Images: A Robust SqueezeNet-Enhanced Machine Learning Framework

**DOI:** 10.1155/ijta/2257215

**Published:** 2025-02-20

**Authors:** Vineet Mehan

**Affiliations:** AI&ML Department, NIET, NIMS University, Jaipur, Rajasthan, India

**Keywords:** acute lymphoblastic leukemia, automatic classification, histopathological images, ML models, SqueezeNet

## Abstract

The growing prevalence of acute lymphoblastic leukemia cancer worldwide underlines the critical need for early and more precise detection to counter this deadly disease. This study presents a robust SqueezeNet-enhanced machine learning framework for automatically screening and classifying histopathological images for acute lymphoblastic leukemia. This work employs a deep learning (DL)–based SqueezeNet integrated with three machine learning (ML) models including neural network (NN), logistic regression (LR), and random forest (RF) for diagnosis. Combining DL and ML algorithms addresses the complexity of understanding histopathological images and the classification process. Evaluation metrics computed for acute lymphoblastic leukemia reveal a good classification accuracy (CA) of 99.3%. Results are further validated by confusion matrix (CM), calibration plot (CP), receiver operating characteristic (ROC) analysis, and comparative analysis with previous techniques. The proposed method has the potential to transform healthcare with more accurate diagnosis. It provides a robust framework for the classification of acute lymphoblastic leukemia, facilitating timely treatment options for patients.

## 1. Introduction

Acute lymphoblastic leukemia (ALL) is a type of blood and bone marrow (BM) cancer [1, 2]. It affects the white blood cells (WBCs) of the body. The rate at which this cancer spreads is very high. Although ALL can happen at any age, it is commonly found in children in the age group of 2–5. In adults, ALL is seen in the age group of 30–50.

BM is the place in the body where stem cells are generated. It is from these stem cells that different blood cells like red blood cells (RBCs), WBCs, and platelets are created. RBCs are also termed as erythrocytes. Similarly, WBCs are also named as leukocytes. A type of leukocyte that is necessary for immunity is lymphocytes. Further, these lymphocytes are of two types: B cells and T cells. B cells originate in the BM whereas T cells originate in the BM but mature in the thymus. The primary role of B cells is to produce antibodies whereas the role of T cells is to kill infected cells. In ALL, immature lymphocytes are created called lymphoblasts [3] also called as leukemic blasts [4]. These lymphoblasts crowd the healthy blood cells and disturb the normal production of RBCs, WBCs, and platelets.

There could be several reasons for the occurrence of ALL which include exposure to radiations [5], like some nuclear accidents, exposure to certain chemicals like benzene, presence of viruses like Epstein–Barr virus (EBV) [6] and human T cell leukemia virus (HTLV) [7]. Environment, family history, mutation of genes, weak immune system, and twin siblings are few other reasons for the occurrence of ALL. These factors increase the risk of development of ALL.

Various symptoms of ALL include bone and joint pain due to the presence of lymphoblasts [8], fever which shows the signs of infection, fatigue that happens due to loss of RBCs, frequent infections due to lack of WBCs, bleeding due to shortage of platelets, enlarged lymph nodes due to infiltration of leukemic blasts, and weight loss as lymphoblasts burn a lot of energy while growing and dividing rapidly.

Various diagnosis methods for ALL [9, 10] include complete blood count (CBC) which is aimed at identifying the count of WBCs, RBCs, and platelets. Higher or lower presence of any type of cells helps in diagnosing the presence of lymphoblasts. BM biopsy is another way for diagnosing lymphoblasts. In this process, a needle is inserted in the pelvis or breast bone to take sample of the BM. The presence of more than 20% of lymphoblasts indicates the occurrence of ALL. Spinal fluid test (SFT) is yet another way for diagnosing ALL. This test is done to check the presence of cancer in the spinal fluid that surrounds the brain and spinal cord. Flow cytometry and immunophenotyping are used to count the subtypes of lymphocytes, that is, B cells and T cells. If the percentage of these cells is higher or lower than the normal, then it could lead to presence of ALL. Cytogenetic and molecular normal tests can be used to check the abnormality in chromosomes and genetic mutations leading to ALL.

ALL patients must eat a healthy and nutritious diet [11] so as to fight the disease effectively. A diet rich in vitamins, minerals, and fiber should be taken which include plants, vegetables, and whole grains [12]. Protein diet like plant-based proteins, cheese, fish, and lean meats is good for health. Healthy fats like nuts, olive oil, and avocados can be taken. Keeping oneself hydrated is always good for health. Alternatively, a diet chart can be followed from a nutritionist in consultation with the doctor treating ALL.

Treatment for ALL includes the most common and effective technique, that is, chemotherapy [13]. This technique is done in three phases which include induction, consolidation, and maintenance. High medicine dose is given in the induction phase, extended dose is given in the consolidation phase, while low dose is given in the maintenance phase. Another way for treatment of ALL is targeted therapy [14] in which medicine is given targeting a specific abnormality present in leukemia cells. BM transplant is another treatment method in which healthy stem cells are induced in the patient from a matching donor. Radiation therapy [15] is another way for treatment of ALL in which high-energy waves are made to enter the human body in order to kill the cancer cells. One or more treatment techniques can be combined depending on the age, condition, and other vital statistics of the patient. Supportive care like nutritional support, blood transfusion, and medicine to manage other infection can also aid in the treatment of ALL.

Recent advancements in the identification of ALL have largely relied on the integration of imaging techniques, machine learning (ML), and artificial intelligence (AI) algorithms. Traditional methods for ALL detection, such as microscopy and manual examination of blood smears, depend heavily on expert interpretation, which can be time-consuming and prone to human error. Current mainstream methods include image-based classification of blood smears using convolutional neural networks (CNNs) and flow cytometry data analysis with ML techniques. These methods have significantly improved the speed and accuracy of ALL diagnosis. For instance, CNN-based approaches have achieved an accuracy of over 90% in automated lymphoblast detection, reducing dependency on manual analysis.

However, existing research methods have certain limitations. Many ML-based models require extensive labeled datasets for training, which are often scarce in medical imaging. Additionally, current methods sometimes struggle with generalization across diverse datasets due to variability in imaging techniques and patient demographics. Furthermore, the interpretability of deep learning (DL) models remains a challenge, making it difficult for clinicians to trust and validate automated predictions.

This paper is aimed at addressing these shortcomings by proposing an optimized multimodel framework for ALL identification, combining SqueezeNet for feature extraction, advanced ML techniques, and ensemble learning. The proposed approach not only enhances the model's accuracy and robustness but also ensures greater interpretability through the integration of the latest AI techniques. This study sets the foundation for a more efficient, reliable, and scalable diagnostic tool for ALL detection.

The paper is organized by giving introduction in Section 1. Literature survey is presented in Section 2. SqueezeNet-enhanced ML paradigm is specified in Section 3. The anticipated methodology and workflow are shown in Section 4. Experimental outcomes are depicted in Section 5. The concluding remarks are given in Section 6. Finally, the references are listed in the end.

## 2. Literature Survey

ALL has been extensively studied in the literature, focusing on its clinical characterization, diagnosis, and classification methodologies. This section organizes and critically analyzes existing research, highlights advancements, and identifies gaps in the current studies.

### 2.1. Clinical Perspectives on ALL

Larson et al. [16] provided a foundational understanding of ALL by identifying that immature cells in the BM lead to its development. They emphasized that once these cells enter the bloodstream, multiple organs, including the nervous system, liver, spleen, and skin, can be affected. This work laid the groundwork for understanding the systemic impact of ALL. Malard and Mohty [17] further explored the progression of ALL, noting that the cancer spreads within 1–4 years. Their analysis revealed that while the disease predominantly affects healthy individuals, a smaller percentage of cases arise due to hereditary or environmental factors. They also observed that recovery rates are higher in children and younger individuals compared to those above 40 years of age.

### 2.2. Classification Models for ALL

Devi et al. [18] proposed a classification model for ALL that accounts for acute and chronic parameters. Their work highlighted the challenges of misdiagnosis due to varying signs and symptoms, achieving a commendable accuracy of 97.85%. Jiwani et al. [19] introduced a pattern recognition system based on DL to identify the stage of ALL. Their findings confirmed that immature WBC counts surpass mature WBC counts in ALL patients. This study highlighted the prevalence of ALL in children and emphasized that recovery rates are generally positive in this demographic.

### 2.3. Advancements in DL-Based Models

Das et al. [20] employed a transfer learning approach using ResNet DL architecture combined with an orthogonal softmax layer. Dropout layers and rectified linear unit (ReLU) layers were incorporated to enhance model efficiency, with comparative analyses showcasing its superiority over previous models. Himel et al. [21] developed an ensemble model combining two DL architectures to improve training and overall efficiency. By integrating dropout layers, the computational speed was significantly enhanced, achieving an impressive accuracy of 99.3%.

### 2.4. Automation and Feature-Based Models

Agaian et al. [22] proposed an automated screening system for acute myelogenous leukemia, aiming to reduce manual labor and diagnostic time. Their model, tested on a dataset of 88 images, achieved an accuracy of 98%, demonstrating the potential of automation in hematological diagnoses. Hossain et al. [23] designed an AI model for ALL classification using datasets from two hospitals. Sixteen key features were identified in collaboration with medical experts. Decision trees and the Apriori algorithm were employed to generate rules, resulting in an accuracy of 97.45%. Sipes and Li [24] introduced an automated CNN for ALL classification. Their work focused on replacing slow, manual techniques with a fine-grained, computationally efficient approach. Comparative analyses revealed significant improvements over traditional methods.

### 2.5. Gaps and Opportunities in ALL Classification

Despite substantial progress, the field still lacks a standardized method for ALL classification. Existing models often face challenges such as limited datasets, variability in diagnostic features, and a lack of generalizability across diverse populations. Additionally, while DL models have shown high accuracy, their computational complexity and resource requirements remain a concern. Moreover, many studies do not adequately address the integration of clinical expertise into the modeling process.

### 2.6. Direction of This Study

This paper is aimed at addressing these gaps by proposing a novel SqueezeNet-enhanced ML paradigm. The proposed methodology leverages the strengths of lightweight architectures, ensemble learning, and domain-specific feature engineering to improve the accuracy and efficiency of ALL classification. This approach seeks to overcome the limitations of existing methods and establish a more standardized framework for ALL diagnosis.

In spite of the substantial work given by many researchers in ALL classification, still a standardized method is deficient in this field of study. In the subsequent section, we will investigate the DL and ML models along with the types of ALL classified.

## 3. SqueezeNet-Enhanced ML Paradigm

In the proposed work, we will use SqueezeNet with three different ML models. SqueezeNet is a CNN with high accuracy for image classification [25]. It has a smaller number of parameters and takes less storage space. Here in this work, SqueezeNet will be used to identify features for four different classes of ALL.

**Figure 1:**
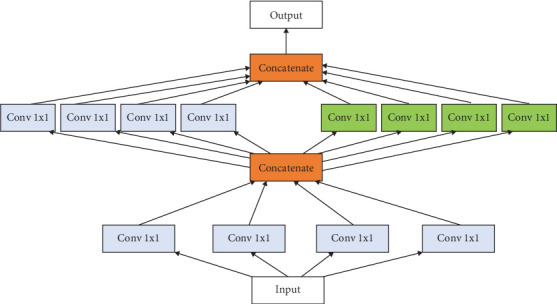
SqueezeNet architecture [26].

The network structure of SqueezeNet includes fire modules. Fire modules used in SqueezeNet make it fast and efficient for classification of cancer. Each fire module consists of two layers: squeeze layer and expand layer. The squeeze layer makes use of 1 × 1 convolutions and thus reduces the input channel. The expand layer uses both 1 × 1 and 3 × 3 convolutions to expand the output channel. SqueezeNet architecture is given in Figure 1. Pruning, quantization, and Huffman coding compress the model size further. The model is useful even with reduced hardware requirement like in mobile devices and embedded systems. The output obtained from SqueezeNet is passed to neural network (NN) [27], logistic regression (LR) [28], and random forest (RF) [29] for training of the models.

In NN, the labeled data with feature identification coming from SqueezeNet is fed into the input layer. Weights, bias, and activation functions are added to it. Further processing is done by hidden layers and finally the output is obtained from output layer. Hyperparameters are fine-tuned in order to achieve a good classification accuracy.

In LR, sigmoid function set to a particular threshold is used for classification purpose. Value below the threshold is assumed to belong to a particular class, while a value above the threshold belongs to the other class. In case of multiclass classification, softmax function [30] is used for the classification which is defined in Equation (1):

(1) $$\begin{array}{c} P\left(y=c/x\right)=\frac{e^{wc.x}}{\sum_{k=1}^{K}e^{wk.x}}P \end{array}$$

where *y* is the predicted class, *x* is the input feature vector, *w*_*c*_ is the weight vector for class *c*, and *K* is the total number of classes. A multiclass classification for the four classes of ALL, that is, Benign, Early, Pre, and Pro, will be computed in the following way.

1. Compute the scores *S*_*B*_ = *W*_*B*_.*x*, *S*_*E*_ = *W*_*E*_.*x*, *S*_*P*_ = *W*_*P*_.*x*, *S*_*O*_ = *W*_*O*_.*x*, where *S*_*B*_ is the computed score for the Benign class, *S*_*E*_ is the computed score for the Early class, *S*_*P*_ is the computed score for the Pre class, *S*_*O*_ is the computed score for the Pro class, *W*_*B*_ is the weight vector corresponding to the Benign class, *W*_*E*_ is the weight vector corresponding to the Early class, *W*_*P*_ is the weight vector corresponding to the Pre class, *W*_*O*_ is the weight vector corresponding to the Pro class, and *x* is the input feature vector.
2. Apply the softmax function for each class [30]:

(2) $$\begin{array}{c} P\left(\frac{B}{x}\right)=\frac{{\text{e}}^{\text{SB}}}{{\text{e}}^{\text{SB}}+{\text{e}}^{\text{SE}}+{\text{e}}^{\text{SP}}+{\text{e}}^{\text{SO}}}, \end{array}$$

(3) $$\begin{array}{c} P\left(\frac{E}{x}\right)=\frac{{\text{e}}^{\text{SE}}}{{\text{e}}^{\text{SB}}+{\text{e}}^{\text{SE}}+{\text{e}}^{\text{SP}}+{\text{e}}^{\text{SO}}}, \end{array}$$

(4) $$\begin{array}{c} P\left(\frac{P}{x}\right)=\frac{{\text{e}}^{\text{SP}}}{{\text{e}}^{\text{SB}}+{\text{e}}^{\text{SE}}+{\text{e}}^{\text{SP}}+{\text{e}}^{\text{SO}}}, \end{array}$$

(5) $$\begin{array}{c} P\left(\frac{O}{x}\right)=\frac{{\text{e}}^{\text{SO}}}{{\text{e}}^{\text{SB}}+{\text{e}}^{\text{SE}}+{\text{e}}^{\text{SP}}+{\text{e}}^{\text{SO}}}. \end{array}$$

3. Class with the highest probability is chosen as the predicted class.

The third ML model used in the proposed work is RF. This model uses a number of decision trees in order to make the prediction. Classification of all four cases of ALL is done in RF. Computational complexity is enhanced while using RF but at the same time there is enhancement of accuracy.

## 4. Methodology

### 4.1. Dataset

**Figure 2:**
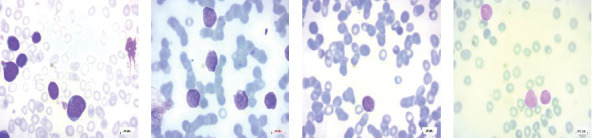
Categories of histopathological images [32]. (a) Benign. (b) Early. (c) Pre. (d) Pro.

The dataset used in the research work is of Taleqani Hospital in Iran. The dataset is freely available for research purpose in public domain [31]. The dataset includes 3256 peripheral blood smear images for 89 patients. A subset of 400 images is taken from the dataset for the experimentation purpose. One image from each category of histopathological images is given in Figure 2. ALL classes include Benign, Early, Pre, and Pro. In each class, 100 images are taken, making the dataset balanced in terms of class distribution. Benign cells are the ones that are healthy immature lymphocytes going through the normal development stage. Early cells are the ones which have started to grow. Pre cells are the one that have matured partially. Pro cells are the one that have matured completely.

Preprocessing techniques applied to the dataset include normalization and resizing. In normalization, scaling of image data is done in the range of [−1, 1] ensuring consistency and making the data suitable for model training. Additionally, all the images are resized to 224 × 224 pixels to standardize the input dimensions of the model.

### 4.2. Experimental Workflow (EW)

**Figure 3:**
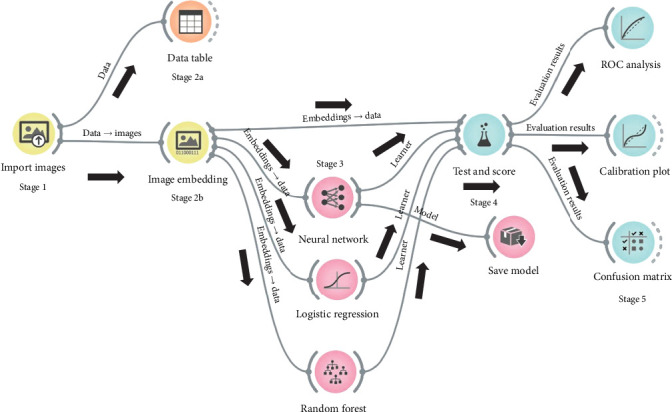
Experimental workflow.

EW for the anticipated research is specified in Figure 3. A unified method of DL and ML for automated classification of histopathological images is specified. Learning from numerous shapes, structure, and features present in histopathological images is done using SqueezeNet DL algorithm. The outcome of the algorithm is passed to NN, LR, and RF classification techniques. Output so obtained is further judged by means of confusion matrix (CM), receiver operating characteristic (ROC) analysis, and calibration plot (CP). The entities in EW represent widgets in Orange Software. A solid line indicates that a widget has an input, while a dotted line signifies that a widget lacks an output.

Various stages of EW include the following:

1. In Stage 1, ALL histopathological images are imported from four subfolders.
2. In Stage 2a, all the 400 images are reflected in a table. There are 400 instances with no missing data, a target with 4 values and 5 meta-attributes.
3. In Stage 2b, image embedding is implemented using SqueezeNet. All the features present in the images are converted into vector form. One thousand different numerical subcategories of features are extracted in this step that accounts to 400,000 numerical representations.
4. In Stage 3, vector of numerical representations is passed to NN, LR, and RF for classification of histopathological images.
5. In Stage 4, test and score evaluation is done with a 10-fold stratified cross-validation and random sampling with repeat train/test of 20.75% of data is reserved for training and the rest for testing.
6. In Stage 5, CM, CP, and ROC analysis are done so as to obtain the final evaluation statistics.

For the model training of NN, several key hyperparameters and configurations were used which include number of hidden layers to be 2 with a size 50 neurons each; activation function used is ReLU for ensuring nonlinearity; learning rate is controlled by L-BFGS-B for smoother convergence of second-order optimization tasks that adapt to the learning rate automatically; regularization *α* is 0.0001; maximum number of epochs taken is 250 to ensure model convergence; replicable training is incorporated for the model for ensuring consistency in results by fixing the random seed. Since the interpretability is critical in ALL cancer, no regularization was incorporated for LR, to prevent the risk of overfitting. Number of trees taken in RF is 10, while the split size of trees is no smaller than 5. Bias in any ML model can lead to inaccurate predictions and unfair outcomes. To ensure fairness, random sampling is used for training with 75% of the data, while stratified sampling is applied for testing and cross-validation with 10-folds.

## 5. Experimental Findings

### 5.1. Evaluation Results

The evaluation results, summarized in Table 1, highlight the performance of various models based on multiple metrics. Among the models, RF achieves the shortest training time (TT) of 4.110 s, while NNs exhibit the highest TT of 11.171 s. For testing time (TET), LR demonstrates the fastest performance at 2.151 s, whereas NN shows the longest TET of 5.205 s.

Despite the higher computational time required for training and testing, NN outperforms other models in terms of predictive performance. It achieves a perfect area under the curve (AUC) value of 1, signifying its exceptional ability to distinguish between classes without error. Additionally, NN delivers the highest classification accuracy of 99.3%, reflecting its near-flawless capability in making correct predictions.

Further metrics, including the *F*1 score, precision, and recall, all at 99.3%, underscore the balanced performance of NN in identifying true positives (TPs) while minimizing false positives (FPs) and false negatives (FNs). Moreover, the Matthews correlation coefficient (MCC) for NN, recorded at 99.1%, indicates a robust and balanced evaluation of the model's classification capability by incorporating all elements of the CM.

These results collectively highlight NN's superior performance across key metrics, signifying its stability and reliability as a classification model. Although NN demands more computational resources due to its higher TT and TET, this trade-off is justified given its outstanding predictive accuracy and robustness. Consequently, NN emerges as the most effective model for this classification task, providing reliable and consistent outcomes.

### 5.2. Comparative Analysis

**Figure 4:**
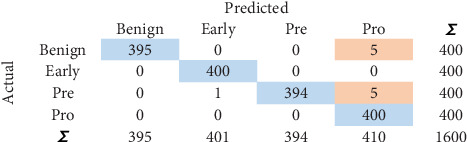
Confusion matrix for NN.

Comparative analysis of the proposed approach with respect to former methods is given in Table 2. Our approach is showing 1.46% and 3.23% increase in CA when compared with previous methods like DL4ALL and ResNet18. Similarly, our approach is showing 0.51% and 1.43% increase in sensitivity. Similarly, our approach is showing 3.51% and 5.58% increase in specificity.  Figure 4 depicts the comparative analysis in the form of a chart.

### 5.3. CM

CM is a *n* × *n* matrix with TP, true negative (TN), FP, and FN values. Diagonal values moving from top left to bottom right give the count of total values that are predicted correctly. CM identified for NN is shown in Figure 4. One thousand five hundred eighty-nine instances show the correct predictions. Similarly, 11 instances show the incorrect predictions.

**Figure 5:**
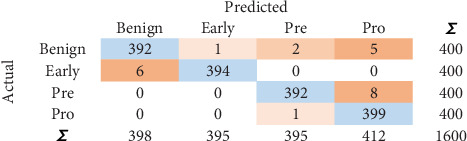
Confusion matrix for LR.

CM identified for LR is shown in Figure 5. One thousand five hundred seventy-seven instances show the correct predictions. Similarly, 23 instances show the incorrect predictions.

**Figure 6:**
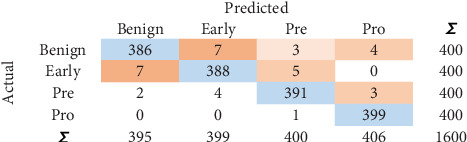
Confusion matrix for RF.

CM identified for RF is shown in Figure 6. One thousand five hundred sixty-four instances show the correct predictions. Similarly, 36 instances show the incorrect predictions.

Seeing all the values from the CM, it is well visualized and identified that NN is performing better than LR and RF.

### 5.4. CPs

**Figure 7:**
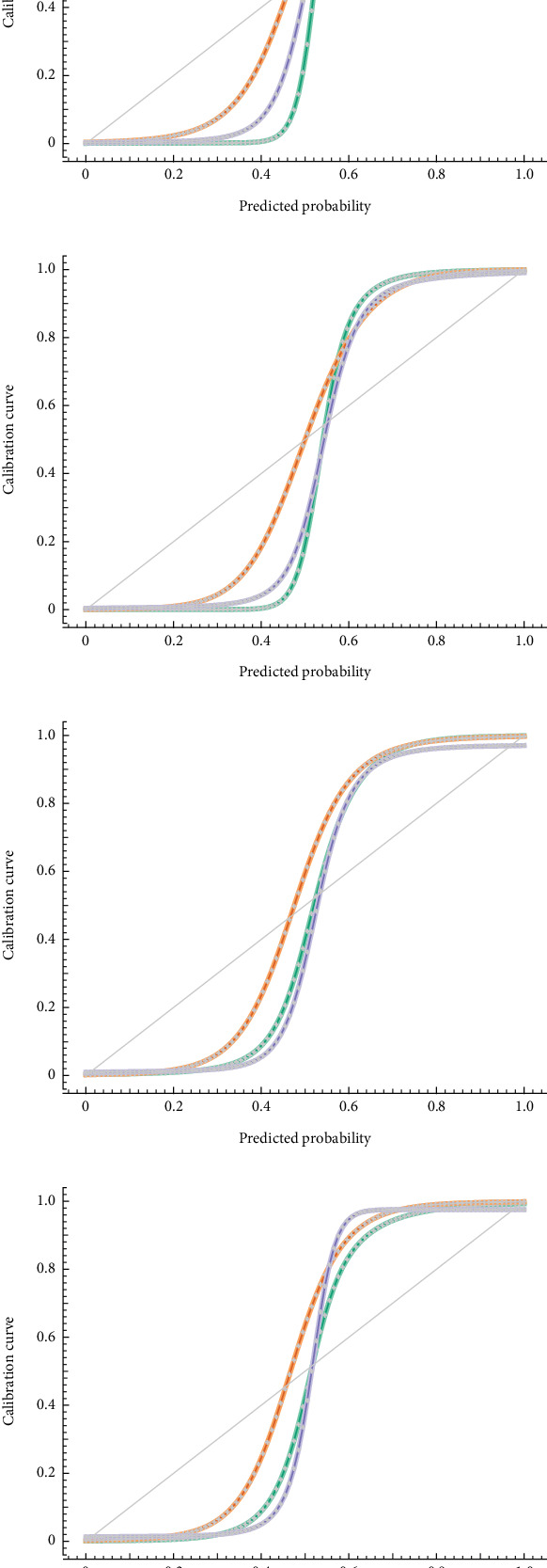
Calibration plots for target classification. (a) CP for Benign. (b) CP for Early. (c) CP for Pre. (d) CP for Pro.

CPs visualize the connection among predicted probabilities and actual results. A well-developed model has an ideal diagonal line. Any line above the diagonal shows the overconfidence of model in prediction. Any line below the diagonal shows the underconfidence of model in prediction. In Figure 7, we see the CP for NN in green, LR in purple, and RF in orange. Seeing the plots, it is clearly visible that NN has the best possible association with respect to the diagonal line in comparison to LR and RF.

### 5.5. ROC Analysis

**Figure 8:**
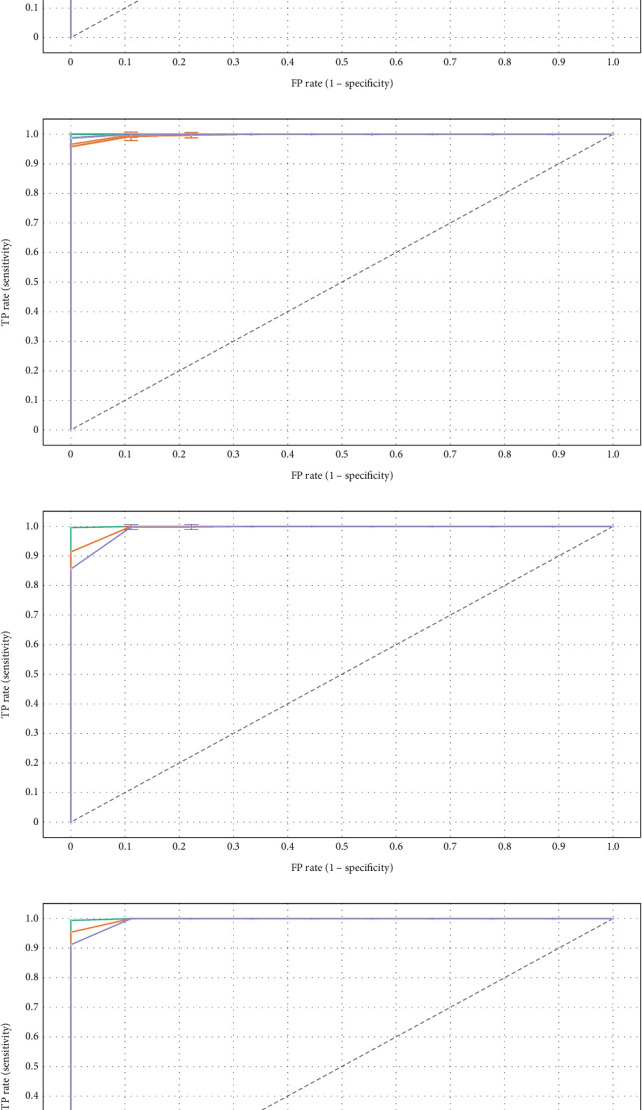
Specificity versus sensitivity. (a) ROC for Benign. (b) ROC for Early. (c) ROC for Pre. (d) ROC for Pro.

ROC analysis is a substantial means that is used to evaluate the ALL classification. It calculates how well the model can distinguish among different classes. Sensitivity and specificity are compared for NN, LR, and RF as shown in Figure 8. ROC curve plots the TP rate on the *y*-axis in contrast to the FP rate on the *x*-axis. The most suitable output lies near the topmost *y*-axis.

The NN achieves the highest AUC, demonstrating superior classification ability compared to LR and RF. This indicates that the NN effectively captures the complex, nonlinear patterns in the dataset, which are crucial for accurate ALL classification.

While LR is efficient in computational terms, its performance is lower, likely due to its linear nature, which restricts its ability to model intricate relationships in the data. The steep initial rise in the curve reflects reasonable sensitivity, but its inability to achieve high TPR at lower FPR values highlights its limitations in this context.

RF shows moderate performance, benefiting from its ensemble structure but falling short of NN's capability. The model may have struggled with overfitting or insufficient tuning, leading to reduced generalization.

NN models excel in handling high-dimensional data with complex structures, which are characteristic of ALL datasets. Advanced feature extraction and optimization techniques in the NN architecture lead to better differentiation between classes. The higher AUC, along with consistent performance across sensitivity–specificity trade-offs, establishes NN as the most reliable model for ALL classification.

## 6. Conclusion

In this paper, we have successfully automated the ALL cancer classification process by SqueezeNet-enhanced ML framework. The proposed approach significantly streamlines the diagnosis process, saves time, and enhances the skills of medical professionals. Integration of SqueezeNet model with NN, LR, and RF is yielding promising results, particularly SqueezeNet with NN which is giving superior results with 99.3% classification accuracy. The investigation results obtained using evaluation parameters, CM, CPs, ROC analysis, and comparative analysis validate the precise performance of the approach. The research significantly enhances the diagnostic capabilities by providing timely and accurate diagnosis. In the future, the model could be enhanced by using a larger dataset to improve generalization, thereby enhancing real-time diagnostic capabilities for clinical deployment.

## Figures and Tables

**Table 1:** Evaluation results.

**Model**	**TT (seconds)**	**TET (seconds)**	**AUC**	**CA**	**F**1	**Precision**	**Recall**	**MCC**
Neural network	11.171	5.205	1.000	0.993	0.993	0.993	0.993	0.991
Random forest	4.110	2.545	0.999	0.978	0.977	0.977	0.978	0.970
Logistic regression	5.104	2.151	0.999	0.986	0.986	0.986	0.986	0.981

**Table 2:** Comparative analysis of the proposed approach with respect to former methods.

**Model**	**CA**	**% increase**	**Sensitivity**	**% increase**	**Specificity**	**% increase**
DL4ALL [33]	97.85	1.46	98.79	0.51	95.81	3.51
ResNet18 [33]	96.09	3.23	97.88	1.43	93.76	5.58
Our approach (SqueezeNet + neural network)	99.3	—	99.3	—	99.3	—

## Data Availability

Data is available for research purpose at [32] which is cited in the paper.
